# Feasibility, Ease-of-Use, and Operational Characteristics of World Health Organization–Recommended Moderate-Complexity Automated Nucleic Acid Amplification Tests for the Detection of Tuberculosis and Resistance to Rifampicin and Isoniazid

**DOI:** 10.1016/j.jmoldx.2022.10.001

**Published:** 2023-01

**Authors:** Anura David, Margaretha de Vos, Lesley Scott, Pedro da Silva, Andre Trollip, Morten Ruhwald, Samuel Schumacher, Wendy Stevens

**Affiliations:** ∗ Department of Molecular Medicine and Haematology, School of Pathology, Faculty of Health Sciences, University of the Witwatersrand, Johannesburg, South Africa; † FIND, the Global Alliance for Diagnostics, Geneva, Switzerland; ‡ National Priority Program, National Health Laboratory Services, Johannesburg, South Africa

## Abstract

Four moderate-complexity automated nucleic acid amplification tests for the diagnosis of tuberculosis are reported as having laboratory analytical and clinical performance similar to that of the Cepheid Xpert MTB/RIF assay. These assays are the Abbott RealTi*m*e MTB and RealTi*m*e MTB RIF/INH Resistance, Becton Dickinson MAX MDR-TB, the Hain Lifescience/Bruker FluoroType MTBDR, and the Roche cobas MTB and MTB RIF/INH assays. The study compared feasibility, ease of use, and operational characteristics of these assays/platforms. Manufacturer input was obtained for technical characteristics. Laboratory operators were requested to complete a questionnaire on the assays’ ease of use. A time-in-motion analysis was also undertaken for each platform. For ease-of-use and operational requirements, the BD MAX MDR-TB assay achieved the highest scores (86% and 90%) based on information provided by the user and manufacturer, respectively, followed by the cobas MTB and MTB-RIF/INH assay (68% and 86%), the FluoroType MTBDR assay (67% and 80%), and the Abbott RT-MTB and RT MTB RIF/INH assays (64% and 76%). The time-in-motion analysis revealed that for 94 specimens, the RealTi*m*e MTB assay required the longest processing time, followed by the cobas MTB assay and the FluoroType MTBDR assay. The BD MAX MDR-TB assay required 4.6 hours for 22 specimens. These diagnostic assays exhibited different strengths and weaknesses that should be taken into account, in addition to affordability, when considering placement of a new platform.

Despite significant progress in the fight against tuberculosis (TB), the disease remains one of the top 10 causes of death worldwide and second only to coronavirus disease 2019 as a leading cause of death from a single infectious agent, ranking above HIV/AIDS.^1^ The most recent threat to the TB cascade of care is the coronavirus disease 2019 pandemic, which re-directed resources, resulting in an approximately 18% decline in the number of patients newly diagnosed with TB and reported compared with 2019.^1^ Of the other gaps and weaknesses identified in the management of TB,^2^ one of the biggest challenges remains the lack of diagnostic and drug resistance detection tools appropriate for different laboratory settings. The introduction of the Xpert MTB/RIF (Xpert) (Cepheid, Sunnyvale, CA), Xpert MTB/RIF Ultra (Xpert Ultra), and the Truenat MTB, MTB Plus, and MTB-RIF Dx assays (Molbio Diagnostics, Goa, India), and their subsequent endorsement by the World Health Organization (WHO, *https://www.who.int/news/item/08-12-2010-who-endorses-new-rapid-tuberculosis-test*, last accessed April 7, 2022; WHO, *https://www.who.int/news/item/25-03-2017-next-generation-xpert-mtb-rif-ultra-assay-recommended-by-who*, last accessed April 7, 2022; and FIND, *https://www.finddx.org/newsroom/pr-02jul20*, last accessed August 1, 2022), represented a substantial improvement in the rapid diagnosis of TB; however, these assays are limited by only providing a rifampicin (Rif) resistance profile. Rif resistance has been considered a reliable indicator of multidrug-resistant TB (MDR-TB) since the WHO estimated that in 2014, only 1.1% of patients with TB worldwide had Rif mono-resistance.^3^ However, in the last few years, there has been increasing evidence that Rif-resistant TB may not be a reliable predictive marker of MDR-TB, especially in countries where higher rates of Rif mono-resistance are being reported.^4^^,^^5^

The new Xpert MTB/XDR (Cepheid) assay may mitigate this limitation because the assay offers isoniazid (INH) resistance detection in addition to the second-line injectable drugs and fluoroquinolones.^6^^,^^7^ However, the lack of Rif and INH resistance detection on a single cartridge is still a disadvantage. The RealT*ime* MTB (RT-MTB) and RealT*ime* MTB RIF/INH Resistance (RT MTB RIF/INH) assays (Abbott Molecular, Des Plaines, IL),^8^ BD MAX MDR-TB assay (Becton, Dickinson and Company, Sparks, MD),^9^ FluoroType MTBDR (FluoroType) assay (Hain Lifescience/Bruker, Nehren, Germany),^10^ and cobas MTB and MTB RIF/INH assays (Roche, Basel, Switzerland)^11^ are therefore attractive options offering *Mycobacterium tuberculosis* complex (MTBC) detection, as well as Rif and INH resistance detection, on a single sputum specimen. An evaluation of these assays involving a laboratory analytical head-to-head comparison^12^ revealed comparable performance of the assays with the Xpert assay for MTBC detection, and with the GenoType MTBDR*plus* assay (Hain Lifescience/Bruker) for Rif and INH resistance detection. Similar clinical performance was reported in a meta-review performed by Kohli et al.^13^

These moderate-complexity automated nucleic acid amplification tests have also been endorsed by the WHO for MTBC and resistance detection (Rif and INH) on respiratory samples.^14^ In addition to assay workflow, several considerations need to be taken into account for laboratory placement. These include: power and space requirements, training and competency, specimen type (raw sputum, sputum pellet, and extrapulmonary specimens), maintenance, quality control and quality assessment, waste disposal, calibration (operator and manufacturer), in-country support, and connectivity (laboratory information systems). To assess these considerations, in addition to the performance data reported,^15^ the feasibility, ease of use, and operational characteristics of these centralized molecular platforms are reported.

## Materials and Methods

For the study, retrospectively collected TB-negative sputum was pooled and homogenized and provided frozen to the study site in 10 mL aliquots. The sputum was spiked with two inactivated, well-characterized MTBC strains in defined stock concentrations (5 × 10^7^ genomes/mL) for the study. These included a high IS6110 copy number strain, MTBC H37Rv, and a low IS6110 copy number strain, *Mycobacterium bovis* Z321. Samples were prepared in a class 2 biosafety cabinet. If controls were not provided by the manufacturer, the un-spiked sputum was then used as a negative control, and commercially available ATCC 25177 (Davies Diagnostics, Johannesburg, South Africa) was used as a positive control (if required).

**Figure 1:**
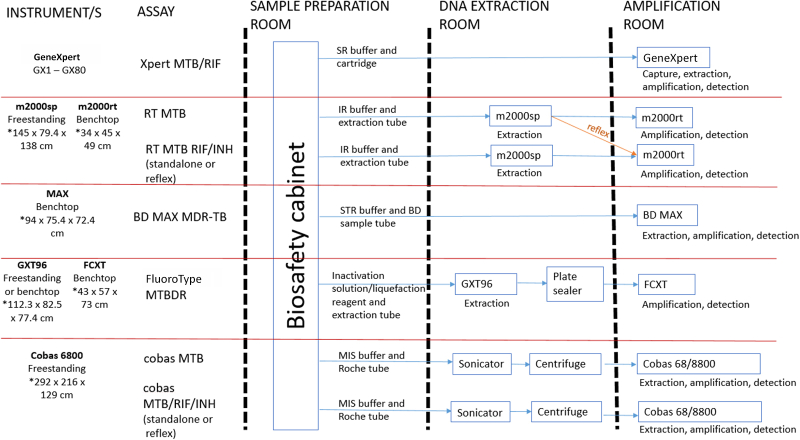
Recommended workflow across assay platforms. **Blue arrows** indicate the sample processing flow for each assay, and the **orange arrow** indicates that DNA extraction does not have to be repeated if reflex testing is performed. FCXT refers to the FluoroCycler XT, GX1-GX80 refers to different sizes of GeneXpert instruments, GXT96 refers to the GenoXtract 96, *m*2000*rt* refers to the Abbott Real-Time PCR instrument, *m*2000*sp* refers to the Abbott Sample Processing instrument, RT MTB RIF/INH refers to the RealT*ime* MTB RIF/INH resistance assay, and RT MTB refers to the RealT*ime* MTB assay. ∗Instrument dimensions are provided as width × depth × height. IR, inactivation reagent; MDR-TB, multi-drug resistant tuberculosis; MIS, microbial inactivation reagent; MTB, *Mycobacterium tuberculosis*; SR, sample reagent; STR, sample treatment reagent.

The feasibility, ease of use, and operational comparative evaluation was performed in the clinical trial laboratory of the Department of Molecular Medicine and Hematology, University of the Witwatersrand (Johannesburg, South Africa). Testing on each platform was performed as per manufacturers’ instructions and guidance and is outlined in Figure 1. Training was provided by application specialists from each of the respective suppliers. Operator competency was assessed as per manufacturer criteria and certification provided before study initiation. A questionnaire on technical requirements of the assay was provided to each manufacturer on the following categories: specimen preparation, reagents, equipment, and support. Questionnaires were completed by technical experts for each manufacturer and reviewed by a senior manager (or equivalent). Based on information received, each characteristic was allocated a score of “1” if shown to be less favorable among the assays and a score of “2” if shown to be more favorable. Category and overall scores were then calculated for each manufacturer.

At study end, each platform operator was requested to complete a Likert scale assessment for each platform [Abbott *m*2000 (order number, 07K25-001), BD MAX (catalog number, BD 441916), Hain Lifescience/Bruker GenoXtract 96 (GXT96; extraction platform; order number, 900044), and FluoroCycler XT (FCXT; amplification and detection platform; order number, 2017] and Roche cobas 6800. A five-point scale was used for each operator to express their level of agreement with each aspect investigated: 1 (very difficult), 2 (difficult), 3 (neutral), 4 (easy), and 5 (very easy). The assessment was performed by at least two operators for each platform, and an average score was assigned for each characteristic. An overall score for each platform was then calculated. In addition, a time-in-motion analysis was performed for each platform. All operational steps, including specimen inactivation and incubation, sonication and centrifugation, reagent reconstitution, platform set-up, and platform testing (including loading and unloading of specimens), were measured. All aspects that should be investigated before assay/platform implementation, except cost, were assessed during the study.

### Abbott RT MTB and RT MTB RIF/INH Assays

Training duration on the *m*2000 system, comprising the *m*2000*sp* for DNA extraction and the *m*2000*rt* for detection and amplification, was 4 days, and an operator was declared competent after three runs. For this study, the inactivation reagent was prepared in-house using the method provided by the manufacturer. Both the positive and negative controls are commercially available from Abbott. The reagent preparation required that absolute ethanol be added to the _m_Wash and _m_Lysis buffers and the addition of an internal control (provided in the amplification kit) to the _m_Lysis buffer. If the correct volume of ethanol was not added to the correct buffer, the liquid sensor detector on the *m*2000*sp* flagged the incorrect volume as an error and paused set-up of the platform until the volume was rectified. MTBC was detected first, and all MTBC-positive specimens could be reflexed for resistance profiling, using the same extracted DNA. If an error occurred during specimen identifier scanning on the *m*2000*sp*, the operator was able to manually enter the specimen identifier or attempt to reposition the tube on the specimen rack and re-scan the tube. If a minor error occurred during processing that did not affect platform functioning, processing continued, and the error code appeared on the result report. For troubleshooting, the error code and possible causes and solutions (for the most commonly occurring errors) could be located in the operator manual, which was available on the *m*2000*sp* and *m*2000*rt* platforms. However, if the error affected the platform’s ability to function, the run was aborted and had to be repeated from the start. Data from the *m*2000*sp* were exported to the *m*2000*rt* (via network transfer or manually using a compact disc).

### BD MAX MDR-TB

One day of training was required on the BD MAX platform, and an operator was declared competent after performing two to three runs. For DNA extraction, each specimen required its own testing strip. All extraction and amplification reagents (except the primers and probes) were included in the testing strip; hence no reagent preparation was required. The tubes containing primers and probes for the assay had to be inserted into the testing strip, which took approximately 1 minute per strip. The tube containing the inactivated specimen as well as the testing strip was slotted into the reagent rack before loading the rack onto the BD MAX instrument. No control material was provided by BD. For amplification, up to 12 specimens were amplified on a single PCR cartridge; hence two PCR cartridges were required for a batch of 24 specimens. MTBC detection and resistance (INH and Rif) profiling were performed at the same time. Once nucleic acid extraction and amplification was completed for a batch of 24 specimens, the next set of 24 specimens could be loaded for testing. If processing on the platform stopped or failed, an error message indicating the cause of the problem was displayed on the screen and could be rectified during repeat testing. Because the 24 specimens were processed in batches of four, if an error occurred during processing, then depending on the severity of the error, testing for a maximum of four specimens had to be repeated. If a noncritical error was encountered during a run, the platform then stopped processing of the affected specimen(s) while testing continued for the unaffected specimens.

### Hain Lifescience/Bruker FluoroType Assay

Use of the GXT96 and FCXT platforms required 3 days of training, and an operator was declared competent after performing three to five runs. Only the positive control was provided in the amplification kit. All reagents required for nucleic acid extraction were provided in bulk volumes and had to be transferred in appropriate volumes to reagent containers designed to fit on the GXT96 platform. The GXT96 software (version 1.0.0.1.0.12) calculated the exact reagent volumes required for the number of specimens to be processed. All reagents were provided in a ready-to-use format except for Buffer B6, which required the addition of an internal control (provided in the amplification kit). If an error occurred on the GXT96, the platform paused the run, displayed the error message, and allowed the run to resume if the error could be rectified. Data from the GXT96 platform were exported to the FCXT platform, thereby creating a run-list on the FCXT platform. The operator also had the option of creating a manual run-list once the run had started.

Hain Lifescience/Bruker was the only manufacturer that recommended an additional autoclaving step of liquid waste prior to disposal. Daily maintenance of the GXT96 took approximately 2 hours because all removable contents (ie, trays and racks) were removed and wiped down with bleach, 70% ethanol, and finally water from a Milli-Q Water Purification System (MilliporeSigma, Burlington, MA). The outer surface of the FCXT platform was cleaned as required.

### Roche cobas MTB and MTB-RIF/INH Assays

The cobas MTB and MTB RIF/INH assays required 5 days for training using the cobas 6800 platform, and an operator was declared competent after performing three to four runs. The inactivation procedure was the most complex of all assays investigated, requiring sonication [using the TS5 (Rinco Ultrasonics, Romanshorn, Switzerland) for 5 minutes at room temperature] and centrifugation (1877 relative centrifugal force for 1 minute) steps, over and above the 60 minutes’ incubation at room temperature. Both controls are commercially available from Roche. If an error was experienced with the specimen identifier reading, the rack containing the specimen was ejected from the platform, which allowed the operator to manually resolve the issue to allow testing to resume. The extraction plate was automatically discarded by the platform once the DNA had been added to the PCR plate for amplification. DNA extraction, therefore, had to be repeated if reflex resistance testing (for Rif and INH) was required. Error information was not provided in the platform manual, and the manufacturer had to be contacted each time the platform displayed an error. If a processing error occurred on the cobas 6800, the entire run had to be repeated. Some errors could be rectified telephonically whereas others required a Roche engineer to rectify on-site.

## Results

### Abbott RT MTB and RT MTB RIF/INH Assays

The inactivation reagent was not provided by Abbott, who recommended an in-country manufacturer or self-preparation. At capacity, 94 specimens and two controls (positive and negative) are tested in a run. Nucleic acid extraction reagents are provided pre-aliquoted in batches of 48 extraction reactions, and amplification reagents are provided pre-aliquoted in batches of 24 tests. If <48 specimens were tested, any residual extraction reagents were discarded. Once opened and if not used in its entirety, amplification reagents could be stored for specified periods of time (RT MTB kit, 14 days at 2°C to 8°C; RT MTB RIF/INH kit, 90 days at −25°C to −15°C) before second use, the latter being controlled by tracking of the first reagent use by the *m*2000*sp* instrument. There was increased operator movement between the rooms and platforms as the amplification plate had to be transferred to the *m*2000*rt* once DNA extraction and PCR set-up was completed on the *m*2000*sp*. Residual DNA (approximately 220 μL) was available once testing was complete. The RT MTB/RIF-INH assay reported on the level of resistance (high level if a deviation in the wild-type pattern was detected in the *katG* gene or low level if a deviation in the wild-type pattern was detected in the *inhA* gene) detected for INH susceptibility; this was in addition to the actual mutation detected in the case of an INH-resistant result or the missing probe (indicating that the wild-type strain was not detected) in the case of a Rif-resistant result. The *m*2000*sp* requires the operator to perform a monthly shutdown and reboot of the platform; the *m*2000*rt* requires a bi-annual optical calibration by the manufacturer or operator.

### Becton Dickinson BD MAX MDR-TB

As opposed to being scanned at the start of testing, the software (version 5.20A) allowed a run-list to be created prior to loading specimens onto the platform (which is a time-saving measure). At capacity, 22 specimens and two controls (positive and negative) are tested in a run. If <24 tests were performed, then any unused wells on a PCR cartridge could be used on subsequent runs. Residual DNA (approximately 10 μL) was available once testing was complete. The BD MAX MDR-TB report contained a Rif and INH resistance profile (if applicable), but no additional information on the detected mutation was provided. The platform produced the least waste (liquid and solid) of all four platforms and requires a bi-annual service and calibration by the manufacturer.

### Hain Lifescience/Bruker FluoroType Assay

Hain Lifescience/Bruker was the only manufacturer that provided panels comprising known positive and negative samples for training and competency. The GXT96 platform was the only platform affected by an increased room temperature (approximately 33°C) and produced incorrect results using the training panel provided. At capacity, 94 specimens and two controls (positive and negative) are tested in a run. Residual DNA (approximately 70 μL) was available once testing was complete. At the time of evaluation, FCXT results could not be interfaced to the laboratory information systems. The FCXT report contained the Rif and INH susceptibility profile (if applicable) and the resistance-conferring mutation (if detected by the assay). Both the GXT96 and FCXT require an annual service, and the GXT96 also requires calibration by the manufacturer.

### Roche cobas MTB and MTB-RIF/INH Assays

At capacity, 94 specimens and two controls (positive and negative) are tested in a run. Because a maximum of five tubes could be sonicated in a batch, it took approximately 120 minutes to sonicate 94 specimens. The results report contained cycle threshold values for Rif and INH resistance, but no additional information on the detected mutation was provided. The loading rack barcode determined the test to be performed on specimens on that rack; thus, the operator had to ensure that the specimen had been loaded onto the correct rack. The built-in refrigerator allowed for on-board storage of reagents (subject to time constraints). Storage of the inactivated specimen was the most advantageous for storage duration for the cobas assays (Supplemental Table S1). The computer interface demonstrated a slow response on the platform’s touch screen. Although the cobas 6800 was also reported to have the most maintenance-related issues, it is an old instrument, and a newer instrument may not experience the similar issues. The platform requires an annual service and calibration by the manufacturer.

### Platform Scores

The BD MAX MDR-TB assay achieved the highest score for each category and overall (Table 1) based on information provided by the manufacturer. The Abbott RT-MTB and RT MTB RIF/INH assays achieved the lowest scores due to difficulties associated with specimen and reagent preparation.

**Table 1:** Allocation of Scores Based on Information Provided by the Manufacturer

Category	Characteristic	Abbott	BD	Hain Lifescience/Bruker	Roche
Specimen preparation	Required specimen type (sputum = 1, sputum and sediment = 2)	2	2	2	2
	Specimen volume (<1 mL = 2)	2	1	2	2
	Specimen may be frozen and thawed prior to processing (= 2)	2	2	1	2
	Specimen preparation is similar for different specimen types (= 2)	2	2	1	1
	No additional consumables/material needed (= 2)	1	1	1	1
	No additional equipment/reagents needed (= 2)	1	2	1	1
	Processed specimen can be used for additional testing (= 2)	1	1	1	1
Reagents	No cold chain shipping is required (= 2)	1	2	1	1
	Reagents expire in <3 months (= 1), or >9 months (= 2) once opened	1	2	2	2
	Room temperature/on-board stability is >1 day (= 2)	1	2	1	2
	Lot to lot testing may be performed (= 2)	1	2	1	2
	No externally sourced reagents are required for the assay (= 2)	1	2	2	2
	Reagents do not need to be loaded onto the instrument at each use (= 2)	1	1	1	2
	TB (or drug resistance) assay reagent does not require reconstitution (= 2)	2	2	2	2
	Positive and negative TB (or drug resistance) controls do not need to be provided by user (= 2)	2	1	1	2
	First use does not affect reagent stability (= 2)	1	2	2	1
	Residual reagents from a kit can be re-used∗ (= 2)	1	2	2	2
Equipment	The platform requires a single space (= 2)	1	2	1	2
	Specimen registration and identification is performed by using a barcode scanner (= 2)	2	2	2	2
	Calculation for tips and reagents is performed automatically (= 2)	2	2	2	2
	The results are provided in PDF, LIS transfer, and electronic copy (= 2) or printout only (= 1)	2	2	2	2
	Information on gene mutation is accessible (= 2), or only the C_T_ value (= 1)	2	1	2	1
	Extracted DNA is available for storage (= 2)	2	2	2	1
	Low potential of DNA contamination of the platform (= 2)	2	2	1	2
	The user does not need to provide a UPS (= 2)	2	2	1	1
	The user does not need to perform data backup manually (= 2)	1	1	2	2
	LIS connectivity is available (= 2)	2	2	2	2
	Remote access for technical support is in place (= 2)	2	2	1	2
	Data can be retrieved/exported through a USB port (= 2)	1	2	2	2
	General laboratory consumables are required to perform maintenance (= 2)	2	2	2	2
	No additional equipment/reagents are required to perform maintenance (= 2)	1	2	2	2
	Instrument calibration is performed at least annually by the manufacturer (= 2)	2	2	2	2
	Instrument calibration kit is provided by the manufacturer (= 2)	1	2	2	2
	The platform is available for multi-purpose testing (= 2)	2	2	2	2
	Different assays can be tested on the same PCR plate (= 2)	1	1	1	2
Support	On-site and remote support is provided (= 2)	2	2	2	2
	A service contract is available (= 2)	2	2	2	2
	In-house training is provided (= 2)	2	2	2	2
	Training requires 1–3 days only (= 2)	1	2	2	1
	Fewer than 3 runs are needed to prove competency (= 2)	1	2	1	1
Overall scores	Specimen preparation	11/14	11/14	9/14	10/14
	Reagents	12/20	18/20	15/20	18/20
	Equipment	30/36	33/36	31/36	33/36
	Support	8/10	10/10	9/10	8/10
	Total	61/80 (76%)	72/80 (90%)	64/80 (80%)	69/80 (86%)

A score of 2 was allocated if the characteristic being investigated was more favorable, and a score of 1 was allocated for a less favorable finding. BD, Becton Dickinson; C_T_, cycle threshold; LIS, laboratory information systems; TB, tuberculosis; UPS, uninterruptible power supply.

∗ For the Abbott assay, this score refers to the extraction kit reagents only.

A heat map is used to represent the score assigned by the platform operators. The gray scale color spectrum demonstrates the least to most favorable responses provided by the operators. The BD MAX MDR-TB was the most favorable of the four platforms, with the Abbott *m*2000 system being the least favorable (Table 2).

**Table 2:** Ease-of-Use Table as Scored by Platform Operators

Category	Characteristic	Abbott			Hain Lifescience/Bruker		
		RT MTB and MTB RIF/INH		BD	FluoroType MTBDR		Roche
		*m*2000*sp*	*m*2000*rt*	BD MAX	GXT96	FCXT	cobas 6800
Training	Training on the use and storage of reagents	2	NA	5	4	4	4
	Training on the use of the consumables	4	4	5	4	4	4
	Training on the use of the software	4	4	5	4	4	3
	Ease of result report interpretation	2	3	5	4	4	4
Reagent and sample preparation	Understand the specimen preparation protocol	2	NA	4	4	NA	2∗
	Ease of specimen preparation	4	NA	4	4	NA	2
	Understand the reagent instructions for use	3†	4	4	3	NA	4
	Reagent storage temperature‡	5	3	5	5	3	4
	Ease of reagent preparation	2	NA	4	2	NA	5
Use of instrument and software	Ease of instrument verification	3	3	3	2	2	3
	Understand the instrument manual	4	4	3	4	4	3
	Understand the device software manual	4	4	4	4	4	3
	Ease of use of the graphic user interface	4	4	4	4	4	3
	Ease of use of the instrument software	4	4	5	4	4	3
	Ease of entering patient identifiers	3	NA	3	2	3	2
	Ease of instrument set-up for a specimen run	2	4	4	2	4	4
	Ease of performing a specimen run	2	4	5	4	4	3
	Ease of performing daily instrument maintenance	2	NA	5	1	NA	4
	Ease of performing weekly instrument maintenance	1	NA	5	2	NA	3†
	Ease of performing monthly instrument maintenance	2	NA	5	3	NA	4
	Minimal reagent wastage and reagent batch size flexibility§	2	NA	5	4	NA	5
	Ease of disposal of solid waste	2	4	5	2	4	3
	Ease of disposal of liquid waste	2	NA	5	2∗	NA	4
Result interpretation	Ease of viewing the result report	NA	4	4	NA	4	4
	Ease in interpreting the result report	NA	2	4¶	NA	4	3
	Ease of exporting the result report	NA	3	4	NA	4	4
	Ease of accessing and viewing previous results	NA	4	5	NA	4	4
Troubleshooting	Understand error codes or flags for run failures	4	4	3	2	2	2
	Ease of troubleshooting frequently occurring errors	3†	3	3	2	2	2
Overall scores	Training	12/20	11/15	20/20	16/20	16/20	15/20
	Reagent and sample preparation	16/25	7/10	21/25	18/25	3/5	17/25
	Use of instrument and software	37/70	31/40	61/70	40/70	33/45	47/70
	Result interpretation	NA	13/20	17/20	NA	16/20	15/20
	Troubleshooting	7/10	7/10	6/10	4/10	4/10	4/10
	Total	72/125 (58%)	69/95 (73%)	125/145 (86%)	78/125 (62%)	72/100 (72%)	98/145 (68%)

A Likert scale was used to assess the user’s experience for each characteristic, scored as follows: 5, very easy; 4, easy; 3, neutral; 2, difficult; and 1, very difficult.

NA, not applicable.

∗ The assigned score (2) is an average score from all operators.

† The assigned score (3) is an average score from all operators.

‡ Ambient temperature = 5, 2°C to 8°C = 4, −20°C (or colder) = 3.

§ Minimal reagent wastage = 5, reasonable reagent wastage = 4, excessive reagent wastage = 2.

¶ The assigned score (4) is an average score from all operators.

### Time-in-Motion Analysis

**Figure 2:**
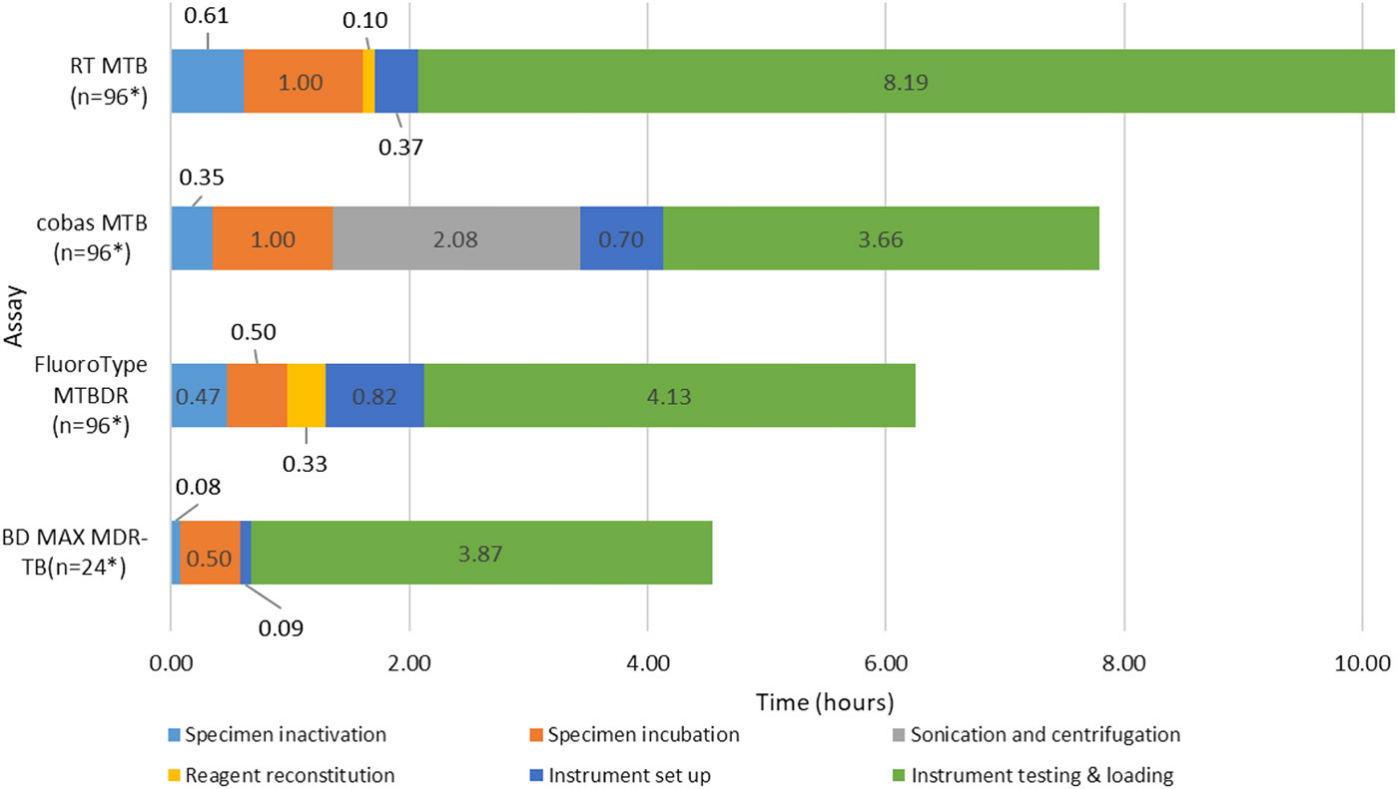
Time-in-motion, at maximum capacity, for all four platforms. RT MTB refers to the Abbott RealT*ime* MTB assay. Numerical values on the bar graphs indicate the time taken for a particular process. ∗Includes that a negative and positive control is included. MDR-TB, multi-drug resistant tuberculosis; MTBDR, *Mycobacterium tuberculosis* drug-resistant assay.

All assays required similar processing steps, with differences in duration. Processing times (before loading the specimens onto the instrument) and instrument run-time for each assay, at capacity, were calculated and are outlined in Figure 2. The Abbott RT-MTB assay required approximately 10.3 hours for 94 specimens, with an additional 2.5 hours required for resistance profiling. The BD MAX MDR-TB assay required 4.6 hours for processing 22 specimens. In terms of hands-on time to process 94 specimens, the Roche cobas MTB and MTB RIF/INH required 3.7 hours, the Hain Lifescience/Bruker FluoroType MTBDR required 2.1 hours, and the Abbott RT MTB and RT MTB RIF/INH assays required 1.9 hours. The hands-on time to process 22 specimens on the BD MAX MDR-TB required 26 minutes.

## Discussion

Rapid, accurate diagnosis of TB and the detection of drug resistance are critical aspects in effective personalized treatment of TB. Point-of-care tests that can be used in primary care facilities are the ultimate goal, but in the interim, the diagnostic pipeline has evolved beyond the Cepheid GeneXpert platform, and several molecular assays for use in central laboratories have been developed and have been recommended by the WHO.^14^^,^^15^ Although the modular format of the GeneXpert technology allows placement of these platforms across the spectrum of laboratories (from point-of-care to core laboratories), the *m*2000, FluoroType, and cobas platforms are more suited to a core laboratory. The BD MAX (due to its smaller footprint) can be placed at the level of a district hospital laboratory. In terms of performance, compared with the Xpert MTB/RIF assay, using spiked sputum, the Abbott RT MTB, BD MAX MDR-TB, and Roche cobas MTB assays displayed increased analytical sensitivity, whereas the Hain Lifescience/Bruker FluoroType assay demonstrated a lower analytical sensitivity.^15^ All four assays showed accuracy comparable to that of the GenoType MTBDR*plus* assay for the detection of Rif and INH resistance.^15^ This study represents a head-to-head laboratory-based comparison on feasibility, ease of use, and operational characteristics of the four new TB diagnostic platforms. Although the platforms exhibit some similarities, each has advantages and disadvantages, which are discussed in the following sections.

### Specimen and Reagent Preparation

The sonication step required for the cobas assays is a limiting step. Although the manufacturer intends to improve the capacity of the sonicator (Roche Molecular Systems Inc., Pleasanton, CA), this process is the reason for operators assigning a low score for specimen preparation. The use of additional sonicators may reduce sample processing time; however, for the purpose of this study, only one sonicator was available. An advantage of the cobas platform is that on-board reagent storage allows continual loading of approximately 350 specimens without the need to replenish reagents between each run. For resistance testing, the same inactivated specimen can be used, provided that sufficient volume is available. The BD and the cobas assays offer the advantage of the entire testing process being performed on a single platform. The BD assay format is also advantageous because all reagents and consumables required for testing are preloaded onto the testing strip with the exception of the tubes containing the primers and probes. However, controls have to be externally sourced for the BD assay, which adds to the cost. The cobas platform is the only one in which DNA extraction has to be repeated if resistance testing is required, which can have time and cost implications in settings with a high incidence of DR-TB. For the Abbott system, a single DNA extract can be used. The BD and FluoroType assays detect MTBC and resistance in the same run, which is an advantage in settings with a high incidence of DR-TB. The Abbott assays are the only assays in which reagent wastage may occur if a maximum number of specimens is not tested, as any unused reagent is discarded if not used within the prescribed times. However, this factor will not present a problem for high-throughput laboratories. Purchase or in-house manufacture of the Abbott inactivation reagent has an additional cost and/or time implication.

### Platform Capacity

The on-board storage function of the cobas 6800 translates into a larger number of specimens being tested in a shorter period of time (384 specimens on the cobas 6800, and 960 specimens on the cobas 8800 in an 8-hour shift). The advantages of this workflow have been previously reported by Aretzweiler et al*.*^16^ The cobas is the only platform that allows testing (of other racks) to continue while a specimen identifier problem is being resolved. However, the cobas 6800 has the longest hands-on times of the high-throughput platforms. The BD MAX platform allows a run-list to be created before specimen loading, the loading of specimens in a random manner, and a second batch of specimens to proceed with nucleic acid extraction while the first batch is being amplified. These time-saving measures make the platform an attractive option for MDR-TB diagnosis, although the full capacity of the platform is only 22 specimens, compared with the other platforms, which process 94 specimens per run. The FluoroType assay has the shortest processing time among the high-throughput platforms, and the FCXT software (version 1.0.1.4.20.62) allows a run-list to be created once the run has started, which is a time-saving advantage. The Abbott assays have the longest processing time of all the high-throughput platforms but the shortest hands-on time. Another factor to consider is that all four platforms are capable of multi-disease testing, and certain combinations of assays can be performed on the same run.^17^

### Platform Requirements

The cobas platforms require the largest space and have special electricity requirements, which are important implementation considerations. The BD MAX achieved the most favorable scores for ease of use and operational considerations; because of its small footprint, multiple BD MAX platforms can be installed in a single room, although this will have cost implications. Disadvantages of the FluoroType assay are that an extra piece of equipment (plate-sealer) is required, which has space and cost implications, and contamination is of concern due to potential spillage while transferring the amplification plate to the plate sealer. Furthermore, liquid waste produced by the GXT96 requires autoclaving before disposal, which has time implications. Daily maintenance is time-consuming, which is an important consideration for workflow in a busy, high-throughput laboratory. The Abbott *m*2000*sp* and *m*2000*rt* platforms cannot be placed in the same room, which presents a challenge to laboratories with space constraints. Daily maintenance is time-consuming, and purchase of an optical calibration kit is required for bi-annual optical calibration of the *m*2000*rt*.

### Reports

The cobas and BD assays do not provide any information on the actual mutations detected. Reports produced on the FluoroType provided the most comprehensive mutation information compared with the other platforms. For the Abbott *m*2000*rt* platform, storage of results requires a compact disc, which is not easily available for purchase and, once stored on the compact disc, a compact disc drive (which is becoming an obsolete technology) is required to read the results. For Rif resistance, the absence of a signal from the corresponding target probe is reported instead of an actual mutation.

### Troubleshooting

The number of errors that occurred on all platforms was minimal except for the cobas 6800, which produced a high number of maintenance-related errors. Because it was difficult to obtain the required error codes to rectify errors, troubleshooting by the operator was reported to be the most complex on this platform.

## Conclusions

In addition to performance and cost, aspects such as the ease of use, operational requirements, assay menu, and feasibility of the assay and platform/s are important when implementation is being considered. This comparative study shows that each platform evaluated has differing strengths and weaknesses. Individual laboratory needs will therefore play a decisive role in the placement of a centralized molecular platform for MTBC and resistance testing.
