# Supplementary material for: Feasibility, Ease-of-Use, and Operational Characteristics of World Health Organization–Recommended Moderate-Complexity Automated Nucleic Acid Amplification Tests for the Detection of Tuberculosis and Resistance to Rifampicin and Isoniazid
Source: J Mol Diagn. 2023 Jan;25(1):46–56. doi: 10.1016/j.jmoldx.2022.10.001 (PMC9830532; doi:10.1016/j.jmoldx.2022.10.001)
Supplement: Supplemental Table S1 [file mmc1.docx]

| **Assay** | **Specimen type** | **Specimen volume** | **Pre-inactivation storage requirements** | | **Post-inactivation storage requirements** | |
| --- | --- | --- | --- | --- | --- | --- |
|  |  |  | **Short-term storage** | **Long-term storage** | **Short-term storage** | **Long-term storage** |
| RT-MTB and  RT-MTB RIF/INH | Raw | 0.3 mL - 10 mL | 2-35°C (≤ 3 days); 2-8°C (Day 4-7) | -25°C to -15°C  (≤ 28 days) | 15-35°C (≤ 48 hours) | n/a |
|  | Sediment | 0.3 mL - 10 mL | 2-8°C (≤ 7 days) | -25°C to -15°C  (≤ 28 days) | 15-35°C (≤ 48 hours) | n/a |
| BD MDR-TB | Raw | min: 1 mL, max: unknown | 2-35°C (≤ 3 days); 2-8°C (Day 4-7) | n/a | 2-8°C (≤ 72 hours) | n/a |
|  | Sediment | min: 1 mL, max: unknown | 2-8°C (≤ 7 days) | n/a | 2-8°C (≤ 72 hours) | n/a |
| FluoroType  MTB-DR | Raw | 0.665 mL - 1.5 mL | 2-35°C (≤ 3 days); 2-8°C (Day 4-7) | n/a | 15-35°C (≤ 2 hours) | n/a |
|  | Sediment | min: 0.5 mL, max: unknown | -20 to -18°C (≤ 1 month) | -80°C to -70°C (≤ 12 months) | 15-35°C (≤ 2 hours) | n/a |
| cobas MTB | Raw | 0.4 mL - 1.2 mL | 2-35°C (≤ 3 days); 2-8°C (Day 4-7) | ≤ -20°C (undefined timeframe) | 2-8°C (≤ 7 days) | ≤ -20°C (30 days) |
|  | Sediment | 0.2 mL - 0.6 mL | 2-8°C (≤ 7 days) | ≤ -20°C (≤ 9 months) | 2-8°C (≤ 7 days) | ≤ -20°C (30 days) |

*Supplemental Table S1: Specimen storage requirements*
